# Modifying the Resistant Starch Content and the Retrogradation Characteristics of Potato Starch Through High-Dose Gamma Irradiation

**DOI:** 10.3390/gels10120763

**Published:** 2024-11-24

**Authors:** Zhangchi Peng, Xuwei Wang, Zhijie Liu, Liang Zhang, Linrun Cheng, Jiahao Nia, Youming Zuo, Xiaoli Shu, Dianxing Wu

**Affiliations:** 1 State Key Laboratory of Rice Biology, Key Laboratory of the Ministry of Agriculture and Rural Affairs for Nuclear-Agricultural Sciences, Zhejiang University, Hangzhou 310058, China; 22116005@zju.edu.cn (Z.P.); 22316211@zju.edu.cn (Z.L.); 22216146@zju.edu.cn (J.N.); 22116004@zju.edu.cn (Y.Z.); shuxl@zju.edu.cn (X.S.); 2 Ningbo Agricultural Technology Extension Station, Ningbo 315000, China; wxwnbcn@163.com; 3 Institute of Crop Science, Jinhua Academy of Agriculture and Technology, Jinhua 321017, China; 18858984660@163.com (L.Z.); clrjh@126.com (L.C.)

**Keywords:** irradiation modification, starch properties, amylopectin structure, paste texture, concanavalin A

## Abstract

Potato starch is widely utilized in the food industry. Gamma irradiation is a cost-effective and environmentally friendly method for starch modification. Nevertheless, there is a scarcity of comprehensive and consistent knowledge regarding the physicochemical characteristics of high-dose gamma-irradiated potato starch, retrogradation properties in particular. In this study, potato starch was exposed to gamma rays at doses of 0, 30, 60, 90, and 120 kGy. Various physicochemical properties, including retrogradation characteristics, were investigated. Generally, the apparent amylose content (AAC), water absorption, gel viscosity, gel hardness, and gumminess decreased as the doses of gamma irradiation increased. Conversely, the resistant starch (RS), amylose content evaluated by the concanavalin A precipitation method, water solubility, and enthalpy of gelatinization were increased. Additionally, swelling power, crystalline structure, and amylopectin branch chain length distribution either remained stable or exhibited only minor changes. Notably, the degree of retrogradation of potato starches on day 7 was positively correlated with the doses of gamma irradiation.

## 1. Introduction

Starch, a major food component, provides structure, texture, consistency, and overall appeal of various food systems [1]. Therefore, starch is an exceptional ingredient within the food industry. According to the treatment it receives, starch is typically categorized as native starch, hydrolyzed starch, and modified starch. Modified starch comprises varieties that have been treated via physical, chemical, or enzymatical methods or combinations of these, resulting in property alterations [2,3,4].

Gamma irradiation is a prevalent, multifunctional, and safe physical modification method in the food industry [5]. It can trigger cross-linking, degradation of starch polymer chains, and other changes by generating free radicals [1,6]. Previous studies have shown that gamma irradiation can break the chains of amylose and amylopectin [1], destroy starch granules [3,7,8], cause cracks on the surfaces of starch granules [9,10], modify the granule surface roughness [11], or change the shape of starch granules [12]. These changes result in the ultimate modifications of the physicochemical properties of starch, including color, water solubility, water absorption, pasting properties, thermal properties, transmittance, crystallinity, freeze–thaw stability, pH, syneresis, carboxyl content, AAC, RS, retrogradation speed, etc. [5,6]. The main purpose of researching gamma irradiation on potato starch is to modify its physicochemical characteristics to satisfy food and industrial utilization [5]. The irradiation doses in most studies were below 30 kGy; thus, investigations into the influences of higher doses on starch properties have not yielded consistent or generalizable results [5,6].

Potato (*Solanum tuberosum* L.) is a traditional staple food and plays a significant role in the food industry [13,14]. Currently, potato is ranked the fourth among the major crops [15]. Fresh potatoes contain approximately 20% dry matter, of which 60–80% is starch; within starch, 47–59 % is classified as resistant starch (RS) [13]. RS is a novel type of functional dietary fiber that is indigestible in the intestines of healthy individuals but can be fermented in the colon by microbiota to produce beneficial short-chain fatty acids [16]. Based on the mechanism of its enzymatic resistance, RS is classified into five subtypes: RS1, RS2, RS3, RS4, and RS5. RS2 mainly exists in raw starch granules, while RS3 is formed as a result of starch retrogradation [13]. Substantial increases in RS content after gamma irradiation (with the maximum dose ranging from 20 to 50 kGy) have been reported [3,9]. However, the RS content of kithul (*Caryota urens*) starch was found to decrease after gamma irradiation [17]. Inconsistent results regarding the effects of gamma irradiation on other physicochemical properties were also reported in similar studies [18]. Starch retrogradation usually brings undesirable impacts on starchy food in the food industry, including reduced shelf life, deterioration of texture, and decreased consumer acceptance [19]. Gamma irradiation may have the potential to modulate the retrogradation rate, thereby influencing the RS content, but may also bring some unexpected changes after further processing.

Thus, potato starch subjected to gamma irradiation at doses exceeding 30 kGy were used as the materials. The starch properties, including RS content, water solubility, thermal properties, pasting, and retrogradation properties, were evaluated. Additionally, the morphology of starch granules and starch structures were investigated. The object of this study is to comprehensively investigate the effects of high-dose gamma irradiation on the RS content and retrogradation of potato starch and to explore the feasibility of modifying starch using high-dose gamma irradiation, which can provide insights for optimizing gamma irradiation treatments to enhance RS content and retrogradation rate of potato starch and explain the mechanism of the effects.

## 2. Results and Discussion

### 2.1. Amylose Content, RS

The apparent amylose content (AAC) was found to be negatively correlated with the gamma irradiation dose (Table 1). Given that short chains are unable to effectively complex with iodine [6], the concanavalin A (Con A) precipitation method was also used to evaluate the amylose content (AC-ConA). The values of AC-ConA for CK, 30, 60, 90, and 120 kGy samples were 43.41, 70.24, 69.77, 71.27, and 71.64, respectively, demonstrating an upward trend as the irradiation doses increased. Meanwhile, the RS content was found to increase along with the increasing irradiation dose (Table 1). Enhancement in the RS content of irradiated starch was also reported by other studies [3,9,20].

The decreases in AAC with the increasing doses are in line with previous studies [3,8,21]. Long-carbohydrate-chain depolymerization resulting in AAC loss was also reported by Shu [20] and Chung [22]. The decrease in AAC can be attributed to the breakage of amylose fraction structure [8,23,24] and long chains in amylopectin caused by gamma irradiation [6]. Short chains cannot complex with iodine effectively [6] and are difficult to detect by the iodine-binding method. The increased AC-ConA after irradiation confirmed the cleavage of long-chain amylose and amylopectin into shorter-chain amylose by irradiation. The appropriate breakdown of starch, along with certain structural modifications and chain cleavage of glycosidic linkages, renders the starch chains in packed matrices inaccessible to digestive enzymes [25], leading to an increase in RS content. Additionally, irradiation can induce the formation of new chemical bonds between anhydrous glucose units through transglucosidation, which further enhances enzymatic resistance [26,27].

### 2.2. Hydration Properties

The water absorption index (WAI) of potato starch decreased from 4.95 (g/g ×100, CK) to 2.11 (g/g ×100, 120 kGy) in a dose-dependent manner. In contrast, the water solubility index (WSI) increased from 21.32 (g/g ×100, CK) to 63.24 (g/g ×100, 120 kGy) as the gamma irradiation dose increased. However, no significant changes were detected in swelling power (SP) (Table 1). Consistent with our findings, numerous studies have reported the enhanced water solubility of potato starch and other starches after irradiation [7,8,9]. However, in contrast to the findings of this study, some researchers discovered that the water absorption capacity of both potato starch [8,10,21] and starch from other plants [9,24] was positively correlated with the irradiation dose. Interestingly, Castanha [28] found that both water absorption and solubility decreased as the irradiation dose increased. Additionally, several reports described a decrease in swelling power or swelling index in irradiated potato starch [8,29], and an opposite opinion of irradiation-increased swelling power was also proposed [21]. It can be hypothesized that the breakdown of long-chain amylose accompanied by the generation of short-chain amylose accounts for the changes in WAI and WSI. This might be because long-chain amylose is capable of absorbing more water, yet it has a lower propensity for hydration compared to short-chain amylose [7,8].

### 2.3. Thermal Properties

With the enhancement in gamma irradiation, the onset temperature (To_1_) and the enthalpy of gelatinization (ΔH_1_) exhibited a rising trend. Specifically, To_1_ rose from 58.07 to 59.60 °C and ΔH_1_ also increased from 16.12 to 17.53 J/g. However, the peak gelatinization temperature (Tp_1_) and the conclusion temperature (Tc_1_) did not exhibit a clear dose-dependent increase, although they did experience an elevation after irradiation (Table 2).

Gelatinization temperature is typically positively correlated with the starch crystallinity and can indicate the stability of starch. In general, irradiation can compromise the stability of starch, leading to a lower gelatinization temperature [11]. A decrease in gelatinization temperatures has been predominantly observed in irradiated potato starch [10]. Nevertheless, in this study, the irradiated potato starch exhibited a higher gelatinization temperature than the native potato starch (Table 2). This might be due to the breakdown of most of the weak crystal structures following radiation exposure [30]. The remaining crystal structures were stronger and required a higher temperature and energy for gelatinization. The increased ΔH_1_ of potato starch after irradiation (Table 2) could imply more robust structures of the remaining starches, which was in line with the observed trend of gelatinization temperature.

### 2.4. Pasting Properties and Gel Texture

As the gamma irradiation dose increased from 0 to 120 kGy, a remarkable reduction trend was discovered in starch viscosity (Table 3). Previous studies have also observed similar impacts of gamma irradiation on the pasting properties of potato starch [3,8,10,29]. Peak viscosity (PV) is primarily associated with the swelling of starch granules. The differences in PV among various potato starches may be due to variations in the swelling index [3,8]. The observed decrease in PV may stem from the disruption of physical bonds within and between starch molecules, which is caused by the altered structural arrangement of starch granules. This damage also leads to a reduction in water absorption ability and swelling of the starch [29]. Setback viscosity (SV) and final viscosity (FV) were primarily influenced by the reorganization or linking of leached amylose and elongated linear amylopectin [3,31]. The decreases in SV and FV after irradiation (Table 3) might be ascribed to the decomposition of amylose and the longer chains of amylopectin caused by irradiation [3].

Simultaneously, as the gamma irradiation dose increased from 0 to 120 kGy, the hardness of the gel significantly decreased from 16.05 to 2.11 N and the gumminess of the gel decreased from 9.16 to 0.64. Additionally, the springiness varied from 0.13 to 0.79 (Table 3). The decrease in paste viscosities might lead to a reduction in hardness [31]. Moreover, it has been reported that a high amylose content is associated with the formation of strong gels [32]. The decreased AAC after irradiation (Table 1) might also contribute to the decreased gel hardness (Table 3). In contrast, the reduced availability of amylose for intermolecular hydrogen bonding disrupts long-range interactions within the gel, resulting in increased aggregates in the absence of a network. This disruption leads to higher stickiness, adhesiveness, and lower cohesiveness [33].

### 2.5. Starch Granule and Crystalline Structure

**Figure 1:**
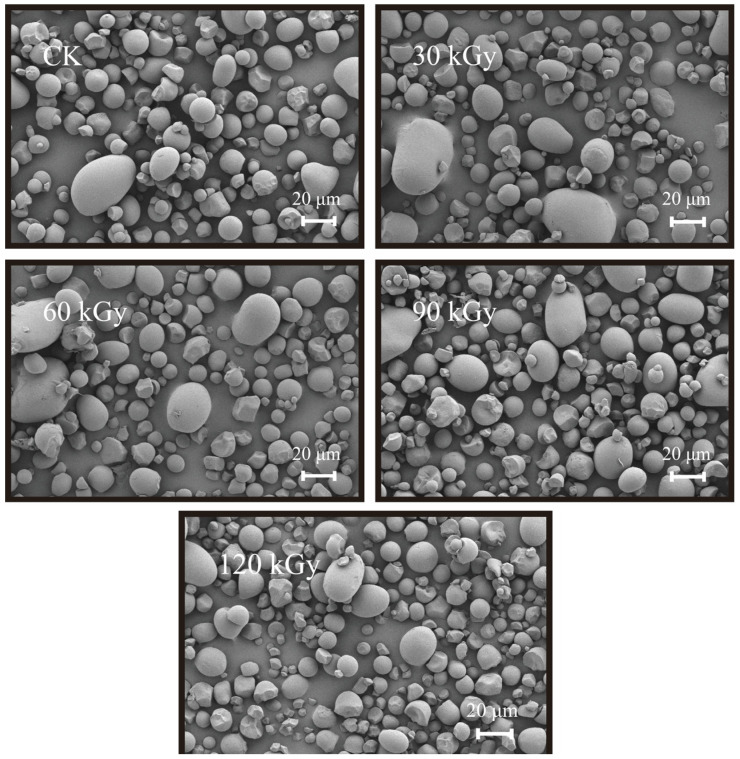
Morphology of native and gamma-irradiated potato starches. CK: native starch, 30 kGy, 60 kGy, 90 kGy and 120 kGy indicated the starch irradiated by 30, 60, 90, and 120 kGy gamma irradiation.

**Figure 2:**
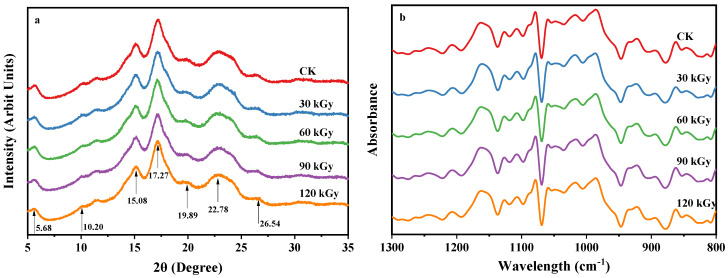
X-ray diffraction (XRD) patterns (**a**) and Fourier transformed infrared (FTIR) spectra (**b**) of native and gamma-irradiated potato starches. The typical hexagonal X-ray diffraction peaks are pointed out by arrows.

The morphology of starch granules showed no visual differences among the various potato starch samples (Figure 1). Furthermore, all potato starch samples exhibited typical B-type starch crystals [34]. No significant alterations were noted in the X-ray diffraction (XRD) patterns, crystallinities, or the Fourier transform infrared (FTIR) spectrums (Figure 2, Table 4). The absorbance ratio of 1042/1024 cm^−1^ can indicate the degree of order in starch granules [31]. The consistent ratio of 1042/1024 cm^−1^ and crystallinity of starch irradiated with different doses suggested that the surface order of starch granules was not influenced by gamma irradiation, even at a dose as high as 120 kGy.

Several studies have also revealed that gamma irradiation exerts no impact on the morphology of starch granules [23,24,31] and crystallinity [7,9,11,17]. However, certain researchers have demonstrated that irradiation can lead to notable alterations in the morphology of starch granules, including cracking, destruction, changes in roughness, surface cracks and fractures, as well as the formation of irregular and polyhedral shapes [3,7,8,9]. Sujka [10] discovered that gamma irradiation led to cracking or destruction of potato starch granules and decreased crystallinity. The discrepancies might stem from variations in starch sources, irradiation doses, and dose rates.

### 2.6. Fine Structure of Amylopectin

**Figure 3:**
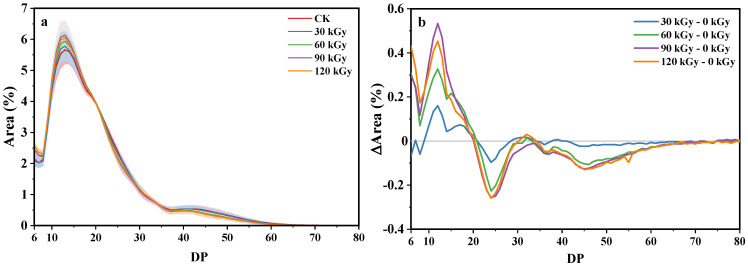
Amylopectin branch chain length distribution of native and gamma-irradiated potato starches (**a**) and the differential chain length distribution of amylopectin between samples irradiated with 0 Gy and other dosages (**b**).

The distribution of amylopectin branch chain length showed variations among different samples. An increase in gamma irradiation doses led to an increase in the short amylopectin branch chain (degree of polymerization, DP 6–20) and a decrease in the long amylopectin branch chain (DP 20–30, DP ≥ 40) (Figure 3), while these differences were not statistically significant when classifying the chains as fa (DP 6–12), fb1 (DP 13–24), fb2 (DP 25–36), and fb2 (DP > 37) (Table 4) as suggested by Nakamura et al. [35]. These findings suggested that gamma irradiation impacted the distribution of amylopectin branch chain length in a dose-dependent manner, with negligible effects at lower doses.

Generally, the average DP of potato starch decreased as the gamma dosage increased from 0 to 50 kGy. During this process, the proportion of short chains increased while that of the long chains decreased [3,6]. Polesi et al. proposed that the cleavage or cross-linking of starch chains triggered by gamma irradiation could be the main factor accounting for the alterations in amylopectin [36].

### 2.7. Retrogradation Properties

After being stored at 4 °C for 7 d, the To_2_, Tp_2_, and Tc_2_ of retrograded starch did not exhibit significant variations among the starches exposed to different irradiation doses, whereas the ΔH_2_ on the 7th day showed a slight increase from 4.27 to 5.75 J/g as the doses increased up to 120 kGy (Table 2). This indicates that gamma irradiation has the potential to enhance the retrogradation of starch.

**Figure 4:**
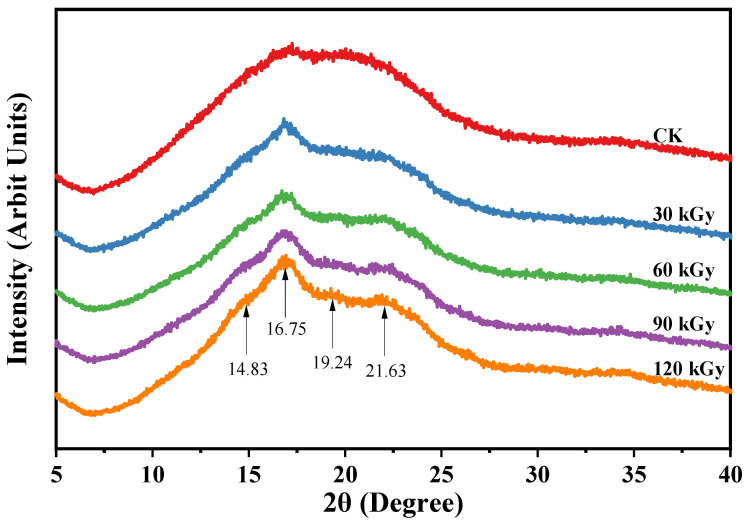
X-ray diffraction (XRD) patterns of gelatinized native and gamma-irradiated potato starches after 7 days of retrogradation at 4 °C.

The increase in ΔH_2_ signified that the retrogradation rate of starch was hastened by gamma irradiation. Usually, retrograded starch displays a typical hexagonal XRD pattern, regardless of whether A- or B-type polymorphs for the native starch [37]. Starch with a higher retrogradation degree shows stronger diffraction peaks [38]. In this study, the XRD patterns of retrograded starches revealed a reconstruction of the hexagonal crystalline structure (Figure 4). Moreover, the diffraction intensity increased with the irradiation dosage (Figure 4). Consequently, it is suggested that the degree of retrogradation on day 7 was positively affected by gamma irradiation. This finding was further corroborated by the calculated relative crystallinity (Table 4). The increased relative crystallinity might imply that gamma-irradiated starches possessed a more ordered long-range structure compared to native starch, as gamma irradiation accelerates the recrystallization of amylopectin [39]. It can also be postulated that the increased short-chain amylose (Table 1) is responsible for the accelerated recrystallization, since amylose molecules recrystallize rapidly in the early stage and then amylopectin recrystallizes more slowly around the nuclei [19,38]. Thus, the initial hardness, stickiness, and digestibility of a starch gel were all affected by amylose retrogradation. However, the alterations in the structure and crystallinity of gelatinized starch were primarily attributed to the retrogradation of amylopectin [19]. High-dose gamma irradiation brings about a decrease in AAC and increases the AC-ConA values (Table 1). These combined effects lead to a more rapid retrogradation process (Table 2). The formation of short chains resulting from the breakdown of long chains, together with the increase in short-chain amylose content due to high-dose gamma irradiation, accelerates retrogradation, which might enhance RS content. Furthermore, the breakdown of amylopectin and the accelerated retrogradation led to a higher degree of recrystallization, making it more difficult for starch-degrading enzymes to access the starch granules.

## 3. Conclusions

Gamma irradiation has a significant impact on the physicochemical properties of potato starch. Firstly, it causes a decrease in apparent amylose content (AAC). However, the AAC method has a limitation as it cannot detect the short-chain amylose. When the amylose contents were assessed with the concanavalin A precipitation method, an increase trend was shown as the irradiation dose increased. Accordingly, it can be hypothesized that the long-chain amylose was broken down into short-chain amylose, which subsequently led to several changes in the physicochemical properties of potato starch. It brought about a decrease in water absorption, gel viscosities (including peak viscosity, setback viscosity, and final viscosity), gel hardness, and gumminess. Meanwhile, it caused an increase in water solubility and the enthalpy of gelatinization. Interestingly, gamma irradiation can accelerate the retrogradation process of potato starches. This phenomenon might also result from the breakdown of long chains. The accelerated retrogradation and recrystallization of potato starch enhances the RS levels, particularly RS3. Nevertheless, further research will be needed to elucidate more specific details, especially regarding the molecular structure and microstructure of starches treated with higher levels of gamma irradiation.

## 4. Materials and Methods

### 4.1. Materials

Potato starch was purchased from Runan Xin’nian Food Co., LTD (Runan county, Zhumadian city, Henan province, PRC) and divided into 5 portions, 1 kg per portion. Subsequently, each portion was irradiated using gamma rays with varying doses (0, 30, 60, 90, and 120 kGy, respectively) at a dose rate of 50 Gy/min. The irradiation process was conducted by Zhejiang Academy of Agricultural Science. All samples were kept at 25 °C under dry conditions.

### 4.2. Determination of Amylose and RS Content

The RS content, including RS2 and RS3, was measured using a modified AOAC method described by Shu [40] but the cooking step was omitted to measure RS2. The apparent amylose content (AAC) was measured using the method described by Bao [41]. Amylose from potatoes (Fluka, 10130, purity 90 %, Honeywell International Inc, Morris Plains, NJ, USA) and amylopectin from maize (Fluka, 10120, purity 99.9 %, Honeywell International Inc., Morris Plains, NJ, USA) were used to construct a standard curve. Meanwhile, the amylose content was also determined with the lectin concanavalin A (Con A) precipitation method reported by Yun and Matheson [42], which is based on amylopectin precipitation. The contents are shown as percentages based on native and irradiated potato flour weight.

### 4.3. Measurement of Water Solubility, Water Absorption, and Swelling Power

The water solubility, water absorption, and swelling power were measured at 80 °C using the method described by Cornejo and Rosell [43] with some modifications.

Briefly, a sample of 100 mg was weighed (m_sa_) into a 2.0 mL centrifuge tube (the weight was recorded as m_b_). Then, 1 mL of ddH_2_O was added. After thorough mixing, the samples were heated at 80 °C for 45 min, with occasional shaking to prevent inhomogeneity. Subsequently, the mixture was centrifuged at 18,400× g for 10 min. The supernatant was collected in a new tube (weight was recorded as m_k_) and the sediment, together with the original centrifuge tube, was weighed immediately (m_se+b_). The weight of the supernatant together with the new centrifuge tube was measured after 2 days of drying (m_su+k_).

The WAI, WSI, and SP at 80 °C are calculated using the following formulas, respectively:

(1) $$\mathrm{Water}\;\mathrm{absorption}\;\mathrm{index}\;\left(\mathrm{WAI}\right)\left(\%\right)=\frac{m_{\mathrm{se}}}{m_{\mathrm{sa}}}\times 100\%$$

(2) $$\mathrm{Water}\;\mathrm{solubility}\;\mathrm{index}\;\left(\mathrm{WSI}\right)\;(\%)=\frac{m_{\mathrm{su}}}{m_{\mathrm{sa}}}\times 100\%$$

(3) $$\mathrm{Swelling}\;\mathrm{power}\;\left(\mathrm{SP}\right)\;(\%)=\frac{m_{\mathrm{se}}}{m_{\mathrm{sa}}-m_{\mathrm{su}}}\times 100\%$$

(4) $$\mathrm{Weight}\;\mathrm{of}\;\mathrm{sediment}\;\left(m_{\mathrm{se}}\right)=m_{\mathrm{se}+b}-m_{b}$$

(5) $$\mathrm{Weight}\;\mathrm{of}\;\mathrm{dissolved}\;\mathrm{solids}\;\mathrm{in}\;\mathrm{supernatant}\;\left(m_{\mathrm{su}}\right)=m_{\mathrm{su}+k}-m_{k}$$

where m_sa_: the weight of the sample; m_se_: the weight of the sediment; m_su_: the weight of the supernatant; m_b_: the blank weight of a 2.0 mL centrifuge tube; m_se+b_: the total weight of a centrifuge tube that contains the sediment; m_k_: the blank weight of a new centrifuge tube that collects the supernatant; and m_su+k_: the total weight of the new centrifuge with the collected supernatant.

### 4.4. Differential Scanning Calorimetry (DSC)

The thermal properties of potato starch were measured with a differential scanning calorimeter (DSC Q200, TA Instruments, New Castle, DE, USA) according to Sun’s method [44] with minor modifications. Briefly, 2.0 ± 0.1 mg of sample was weighed into an aluminum DSC pan, and then 6.0 µL of deionized water was added. The pan was hermetically sealed and equilibrated at 4 °C for 12 h. The samples were then scanned at a heating rate of 10 °C/min from 30 to 110 °C with a hermetically sealed empty pan as a reference. The onset temperature (To), peak gelatinization temperature (Tp), conclusion temperature (Tc), and enthalpy of gelatinization (ΔH) were determined using the analysis tool available in the Universal Analysis software Version 4.5A Build 4. 5. 0. 5 (TA Instruments, New Castle, DE, USA).

### 4.5. X-Ray Diffraction (XRD)

The X-ray diffraction patterns of starches were determined by an X-ray diffractometer (Bruker D8 Advance speed, Bruker AXS, Rheinfelden, Germany) equipped with a copper tube operating at 40 kV and 40 mA, producing Cu Kα radiation of 0.154 nm. Patterns were obtained by scanning from 5° to 40° (2θ) at a step of 0.02°. MDI Jade software V6.5.26 (Materials Data Inc., Livermore, CA, USA) was used to calculate the relative crystallinity [18].

### 4.6. Fourier Transform Infrared Spectroscopy (FTIR)

The potato starch was mixed with KBr powder at a weight ratio of 1:100. Approximately 50 mg of the mixture was pressed into a tablet for FTIR testing. Then, FTIR spectra were obtained using a NICOLET iS50FT-IR spectrometer (Thermo Scientific, Waltham, MA, USA) in the range of 4000–400 cm^−1^ with a 4 cm^−1^ resolution through 32 scans in a transmission mode. Afterward, the raw absorbance–wavelength spectra were deconvoluted over the range of 1300–800 cm^−1^ (half-width: 22 cm^−1^, enhancement factor: 2.2) using Omnic 8.0 (Thermo Fisher Scientific Inc., Waltham, MA, USA).

### 4.7. Rapid Viscosity Analysis (RVA) and Texture Parameters Measurement

The pasting properties of potato starches were tested with a Rapid Visco-Analyzer (RVA-Tecmaster, Perten Instruments, Hägersten, Sweden). Briefly, 2.72 g of flour was weighed and suspended in 25.28 mL ddH_2_O in an RVA canister, making up to a total weight of 28 g. Then, following the procedures described by Bao and Corke [45], peak viscosity (PV), hot paste viscosity (TV), final viscosity (FV), and pasting temperature (PT) were recorded and breakdown viscosity (BV = PV − TV) and setback viscosity (SV = FV − TV) were calculated.

The texture properties of potato starch gel after the RVA test were measured using a TA-XTC-18 texture analyzer (BosinTech, Shanghai, China) with a 5 mm diameter cylinder probe [46]. The pre-speed, post-speed, and test speeds were set as 2 mm/s. The trigger force was set as 0.049 N and the penetration distance was set to 10 mm. Texture parameters, including hardness, gumminess, and springiness, were determined.

### 4.8. Scanning Electron Microscopy (SEM)

The starch granule morphology was investigated by thermal field emission SEM (GeminiSEM 500, ZEISS, Oberkochen, Baden-Württemberg, Germany). The sample was stuck on a specimen holder with a double-sided carbon adhesive tape and then coated with gold under vacuum for 50 s (IB-5 ion coater, Eiko Co., Tokyo, Japan). All samples were observed at an accelerating voltage of 3.0 kV.

### 4.9. Amylopectin Branch Chain Length Distribution

The extraction and debranching of amylopectin were performed according to the method described by Kong [47]. Then, the amylopectin branch chain length distribution was determined by a high-performance anion exchange chromatography (HPAEC) system (Dionex ICS-5000+, Sunnyvale, CA, USA) with a pulsed amperometric detector (PAD) [47]. Maltooligosaccharides with 2−9 DP were used as standard.

### 4.10. Retrogradation

The retrogradation degree of the potato starch gel was assessed utilizing DSC and XRD methods.

The DSC analysis was conducted following the method described by Liu [48] with minor modifications. Briefly, the samples were first scanned, as in Section 2.4; after being stored at 4 °C for 7 days, the samples were subjected to a second scan ranging from 10 to 110 °C. The heat enthalpy changes (ΔH) during the first and second scans were recorded.

For the XRD analysis, 10 g of potato starch was thoroughly mixed with 30 mL of ddH_2_O and heated in a boiling water bath for 10 min. Subsequently, 10 g of the resulting gel were collected immediately, while the remaining gel was preserved at 4 °C for 7 days. The collected samples were then freeze-dried immediately, sieved, and used for XRD analysis as described in Section 2.5.

### 4.11. Statistical Analysis

All experiments were performed in triplicates, except FTIR spectroscopy, which was performed in duplicates. The data were subjected to a one-way analysis of variance (ANOVA) followed by post hoc Duncan’s multiple range tests with SPSS 20.0 (IBM, Armonk, NY, USA).

## Figures and Tables

**Table 1:** AAC, AC, resistant starch content, water absorption index (WAI), water solubility index (WSI), and swelling power (SP) of native and irradiated potato starches *.

Sample	AAC (%)	AC-ConA (%)	RS2 (%)	RS3 (%)	WAI (%)	WSI (%)	SP (%)
CK (0 kGy)	31.96 ± 0.84 ^e^	43.41 ± 0.84 ^a^	45.29 ± 1.67 ^a^	4.73 ± 0.14 ^a^	4.95 ± 0.16 ^a^	21.32 ± 2.09 ^a^	6.29 ± 0.07 ^abc^
30 kGy	27.63 ± 0.38 ^d^	70.24 ± 0.24 ^b^	43.18 ± 0.94 ^a^	5.97 ± 0.42 ^b^	3.32 ± 0.46 ^b^	43.78 ± 5.18 ^b^	5.88 ± 0.30 ^ab^
60 kGy	22.84 ± 0.75 ^c^	69.77 ± 0.50 ^b^	48.35 ± 3.73 ^b^	6.48 ± 0.27 ^c^	2.83 ± 0.37 ^b^	56.68 ± 4.49 ^cd^	6.51 ± 0.17 ^bc^
90 kGy	20.62 ± 0.68 ^b^	71.27 ± 1.19 ^b^	47.95 ± 3.13 ^b^	6.77 ± 0.42 ^c^	3.31 ± 0.14 ^b^	52.27 ± 1.94 ^c^	6.93 ± 0.07 ^c^
120 kGy	16.03 ± 0.78 ^a^	71.64 ± 1.34 ^b^	55.42 ± 0.94 ^c^	7.39 ± 0.31 ^d^	2.11 ± 0.22 ^c^	63.24 ± 0.52 ^d^	5.73 ± 0.53 ^a^

* AAC: apparent amylose content evaluated by iodine staining method, AC-ConA: amylose content evaluated by Con A method, RS2: type 2 resistant starch, RS3: type 3 resistant starch, WAI: water absorption index, WSI: water solubility index, SP: swelling power. The same superscript letters indicate there were no significant differences among samples with different dose irradiation at *p* < 0.05 level.

**Table 2:** Differential scanning calorimetry (DSC) parameters of native and irradiated potato starches *.

Sample	First Scan				Second Scan (7 Days of Retrogradation)			
	To_1_/°C	Tp_1_/°C	Tc_1_/°C	ΔH_1_/(J/g)	To_2_/°C	Tp_2_/°C	Tc_2_/°C	ΔH_2_/(J/g)
CK (0 kGy)	58.07 ± 0.42 ^a^	65.29 ± 0.33 ^a^	79.71 ± 0.40 ^b^	16.12 ± 0.51 ^a^	38.90 ± 0.64 ^ab^	57.15 ± 1.13 ^ab^	73.63 ± 0.29 ^ab^	4.27 ± 0.20 ^a^
30 kGy	58.56 ± 0.13 ^a^	67.35 ± 0.23 ^c^	80.87 ± 0.17 ^c^	16.98 ± 0.28 ^b^	36.71 ± 0.84 ^a^	54.09 ± 1.14 ^ab^	73.02 ± 2.61 ^ab^	5.10 ± 0.06 ^b^
60 kGy	59.21 ± 0.13 ^b^	66.84 ± 0.06 ^b^	78.62 ± 0.29 ^a^	17.01 ± 0.34 ^b^	40.42 ± 2.50 ^b^	57.93 ± 3.14 ^b^	74.32 ± 2.01 ^b^	5.28 ± 0.12 ^b^
90 kGy	59.52 ± 0.25 ^b^	66.55 ± 0.31 ^bc^	80.47 ± 0.14 ^c^	17.21 ± 0.11 ^b^	37.35 ± 0.80 ^ab^	53.21 ± 0.95 ^a^	69.89 ± 0.36 ^a^	6.02 ± 0.28 ^c^
120 kGy	59.60 ± 0.19 ^b^	66.37 ± 0.15 ^b^	80.30 ± 0.45 ^bc^	17.53 ± 0.11 ^b^	38.08 ± 0.60 ^ab^	54.41 ± 0.80 ^ab^	70.15 ± 1.38 ^a^	5.75 ± 0.13 ^c^

* To: onset temperature, Tp: peak gelatinization temperature, Tc: conclusion temperature, ΔH: enthalpy of gelatinization. The subscript number represents which scan this parameter belongs to. The same superscript letters indicate there were no significant differences among samples with different dose irradiation at *p* < 0.05 level.

**Table 3:** Pasting parameters and gel texture properties of native and irradiated potato starches *.

Sample	PV (RVU)	HPV (RVU)	BV (RVU)	FV (RVU)	SV (RVU)	PT (°C)	Hardness (N)	Gumminess	Springiness
CK (0 kGy)	4276.33 ± 26.41 ^e^	2382.00 ± 33.18 ^c^	1894.33 ± 6.94 ^e^	3152.67 ± 49.26 ^c^	770.67 ± 20.00 ^c^	66.52 ± 0.33 ^a^	16.05 ± 1.72 ^d^	9.16 ± 0.58 ^e^	0.6 ± 0.02 ^b^
30 kGy	2040.33 ± 25.98 ^d^	387.67 ± 4.19 ^b^	1652.67 ± 23.30 ^d^	444.67 ± 5.79 ^b^	57.00 ± 1.63 ^b^	69.22 ± 0.02 ^b^	15.65 ± 1.59 ^d^	6.39 ± 0.86 ^d^	0.74 ± 0.03 ^c^
60 kGy	1792.33 ± 14.94 ^c^	214.00 ± 0.82 ^a^	1578.33 ± 15.17 ^c^	232.33 ± 1.24 ^a^	18.33 ± 0.47 ^a^	71.02 ± 0.38 ^c^	12.42 ± 0.86 ^c^	4.20 ± 0.02 ^c^	0.54 ± 0.02 ^b^
90 kGy	481.33 ± 25.38 ^b^	186.67 ± 0.47 ^a^	294.67 ± 25.10 ^b^	199.67 ± 0.94 ^a^	13.00 ± 0.82 ^a^	69.15 ± 0.00 ^b^	5.89 ± 0.88 ^b^	2.08 ± 0.54 ^b^	0.13 ± 0.10 ^a^
120 kGy	322.00 ± 6.53 ^a^	183.33 ± 1.25 ^a^	138.67 ± 5.79 ^a^	193.67 ± 0.94 ^a^	10.33 ± 0.47 ^a^	69.92 ± 0.61 ^b^	2.11 ± 0.13 ^a^	0.64 ± 0.33 ^a^	0.79 ± 0.08 ^c^

* PV: peak viscosity, HPV: hot paste viscosity, FV: final viscosity, PT: pasting temperature, BV: breakdown viscosity (BV = PV − TV), SV: setback viscosity (SV = FV − TV). The same superscript letters indicate there were no significant differences among samples with different dose irradiation at *p* < 0.05 level.

**Table 4:** Crystalline parameters and amylopectin branch chain length distribution of native and irradiated potato starches *.

Sample	Relative Crystallinity ^1^ (%)	Relative Crystallinity ^2^ (%)	FTIR Ratio 995/1024 (cm^−1^)	FTIR Ratio 1048/1024 (cm^−1^)	DP6–12 (%)	DP13–24 (%)	DP25–36 (%)	DP ≥ 37 (%)
CK (0 kGy)	16.63 ± 0.05 ^b^	5.75 ± 0.20 ^a^	1.184 ± 0.040 ^b^	0.829 ± 0.011 ^b^	24.47 ± 2.09 ^a^	51.42 ± 1.57 ^a^	15.10 ± 1.33 ^a^	9.02 ± 2.36 ^a^
30 kGy	17.32 ± 0.03 ^d^	8.40 ± 0.12 ^b^	1.063 ± 0.021^a^	0.787 ± 0.002 ^a^	24.71 ± 2.12 ^a^	51.71 ± 1.42 ^a^	14.98 ± 1.43 ^a^	8.60 ± 2.11 ^a^
60 kGy	16.35 ± 0.06 ^a^	10.62 ± 0.35 ^c^	1.124 ± 0.008 ^ab^	0.800 ± 0.007 ^a^	26.07 ± 1.63 ^a^	52.24 ± 0.92 ^a^	14.45 ± 1.18 ^a^	7.24 ± 1.38 ^a^
90 kGy	16.92 ± 0.14 ^c^	12.35 ± 0.19 ^d^	1.131 ± 0.018 ^ab^	0.809 ± 0.003 ^ab^	26.71 ± 2.10 ^a^	52.24 ± 1.37 ^a^	14.11 ± 1.43 ^a^	6.94 ± 2.04 ^a^
120 kGy	16.54 ± 0.10 ^b^	10.99 ± 0.25 ^c^	1.123 ± 0.005 ^ab^	0.802 ± 0.003 ^a^	26.80 ± 1.43 ^a^	51.93 ± 1.01 ^a^	14.46 ± 0.92 ^a^	6.81 ± 1.53 ^a^

* DP: degree of polymerization. The same superscript letters indicate there were no significant differences among samples with different dose irradiation at *p* < 0.05 level. ^1^: ungelatinized samples. ^2^: gelatinized samples stored at 4 °C for 7 d.

## Data Availability

The data are available from the corresponding author on reasonable request.
